# Neuroretinitis Caused by *Bartonella henselae* (Cat-Scratch Disease) in a 13-Year-Old Girl

**DOI:** 10.1155/2010/763105

**Published:** 2010-06-15

**Authors:** Teodoro Durá-Travé, Maria Eugenia Yoldi-Petri, Fidel Gallinas-Victoriano, Ana Lavilla-Oiz, Marta Bove-Guri

**Affiliations:** ^1^Pediatric Neurology Unit, Children's Hospital “Virgen del Camino”, 31008 Pamplona, Spain; ^2^Ophthalmology Department, Children's Hospital “Virgen del Camino”, 31008 Pamplona, Spain

## Abstract

Cat-scratch disease-related neuroretinitis is a relatively unusual pathology, with suspicious clinical epidemiological and serological diagnosis. We present a case of an adolescent suffering from unilateral neuroretinitis associated with *Bartonella henselae* infection characterized by abrupt loss of vision, optic disc swelling, and macular star exudates with optimal response to antibiotic treatment.

## 1. Introduction

Cat-scratch disease (CSD) is a zoonotic infection caused by the bacillus *Bartonella* *henselae*, which can be transmitted to humans after contact with infected cats. Typical presentation is characterized by a primary lesion (papule) at the site of inoculation (cat scratch or bite) followed by the development of regional painful and/or suppurative lymphadenopathy that is, occasionally, associated to systemic symptoms (fever, discomfort, etc.) and tends to have spontaneous resolution in a few weeks [1, 2]. 

Atypical presentations or disseminated infection, except for immunosuppression, are exceptional, even though cases showing neurological manifestations (encephalitis, facial paralysis, transverse myelitis, etc.), ophthalmologic complications, hepatosplenic granuloma, osteomyelitis, endocarditis, prolonged fever, and so forth, have been described; in these cases, diagnostic suspicion and immediate antibiotic therapy are fundamental [3–8].

We present a case of unilateral neuroretinitis associated to *Bartonella henselae* infection in a teenager with optimal response to antibiotic treatment.

## 2. Case Report

A 13.7-year-old patient was admitted to the emergency department because of vision loss—starting seven days before admission—, ocular pain, and photopsia in the left eye. Symptoms were sudden (at the time she was doing a school test) and coincided with an upper respiratory tract infection with fever in the previous 2-3 days. She had no vomiting and did not complain of headache. There was no trauma history. She had daily contact with cats.

Personal history: Asthma (allergy to dust mites, pollen, and animal epithelium) and strabismus/astigmatismus. Familiar history: she has a sister suffering from idiopathic partial epilepsy, a paternal uncle who suffered a cerebral infarction (cerebral aneurysm), a paternal aunt suffering from multiple sclerosis, and a maternal grandfather who suffered a cerebral stroke.

**Figure 1:**
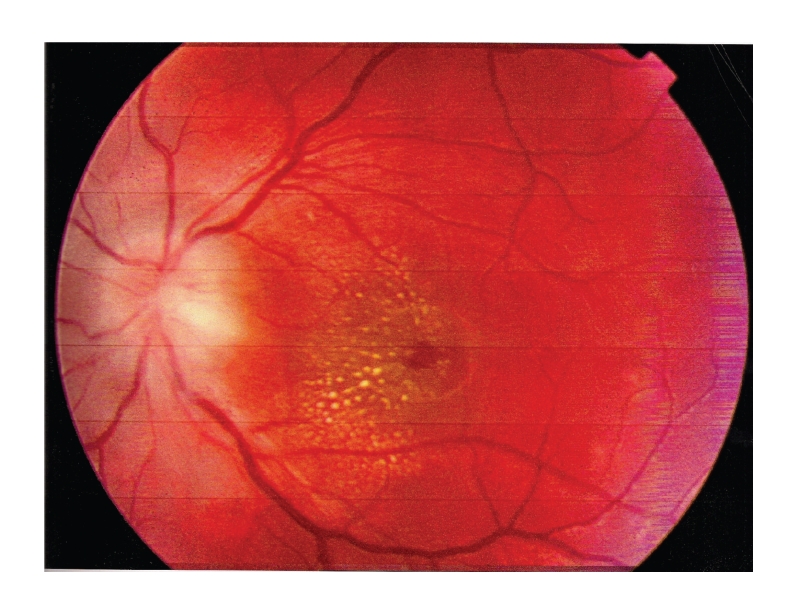
Left eye retinography at the time of admission: papilledema and macular star exudates.

Physical examination showed axillary temperature of 37.8°C. There were not any skin lesions, axillary or inguinal adenopathies noted. The cervical nodes were palpable. Blood pressure was 110/67 mmHg. Ophthalmologic examination revealed a decrease of visual acuity in the left eye (right eye = 0.9, left eye = 0.3), disc swelling and macular star exudates with detachment of neuroepithelium (Figure 1), and central scotoma in campimetry. Blood analysis showed leukocytosis and neutrophilia and an increase of erythrocyte sedimentation rate (ESR = 72 mm) and C-reactive protein (CRP = 12 mg/dl). Biochemistry, proteinogram, coagulation, immunological, antinuclear antibodies, and rheumatoid factor studies were normal. Pressure (170 mmH_2_O), leukocyte count, and protein and glucose in cerebrospinal fluid (CSF) results were normal, and monoclonal bands were not detected. Bacteriological studies (blood and CSF culture) were negative. Serological studies (HSV-I, HSV-II, adenovirus, CMV, EBV, VIH, VDRL, *Mycoplasma pneumoniae*, *Rickettsia conorii*, *Borrelia burdoferi,* and toxoplasma) were also negative, except for antibodies titer to *Bartonella henselae* (indirect immunofluorescence, (IFI)): IgM 1/80 (positive > 1/10) and IgG 1/800 (positive > 1/100). Chest radiography posterior-anterior and lateral projection was normal. Mantoux test was negative. Cranial magnetic resonance imaging (MRI) was normal. Visual evoked potential test showed an increase in latency and a decrease in amplitude in the left eye. Auditory and somatosensory evoked potential tests were normal.

*Progression*. On initial findings (neutrophilia and increase in acute-phase reactants) antibiotic therapy with cefotaxime was started. Several days later (negative bacteriology, visual evoked potential alteration, and normal neuroimaging) oral prednisone was added to her treatment (80 mg/24 hours, 10 days), and after clinical and epidemiological suspicion of neuroretinitis associated to cat-scratch disease (serology was still unknown), treatment with cefotaxime was ended, and rifampicin (300 mg/12 hours) and doxycycline (100 mg/12 hours) were prescribed. This treatment was maintained for 6 weeks after serological confirmation of *Bartonella henselae* infection. The patient remained hospitalized for 15 days, and, by the time of discharge, retinography image and visual acuity had improved (left eye = 0.6), visual evoked potential tests were normal, and antibody titers to *Bartonella henselae* had increased (IgG = 1/1600). After six weeks of antibiotic therapy, ophthalmologic exploration was completely normal, and visual acuity had recovered (left eye = 0.9, right eye = 0.9).

## 3. Discussion

*Bartonella henselae* infection is usually a self-limited and oligosymptomatic disease in immunocompromised patients. It usually manifests as a regional painful lymphadenopathy preceded by an erythematous papule and/or pustule located in the place of a cat scratch or bite. Atypical presentations are quite variable in patients likely to be immunocompromised, and neuroretinitis associated with cat-scratch disease is not frequently seen [1, 5, 9, 10].

Neuroretinitis usually appears a few weeks after typical symptoms of this pathology manifest, which many times goes unnoticed. The main symptom is the abrupt unilateral loss of visual acuity although cases with bilateral affectation have been described [11–13]. The finding of disc swelling associated to macular star exudates, as it happened in this patient, is considered as a predictable sign of an ocular manifestation of cat-scratch disease [9, 14, 15]. However, other etiologies should be discarded as a cause of optic neuropathy, such as arterial hypertension, diabetes mellitus, pseudotumor cerebri, syphilis, tuberculosis, toxoplasmosis, Lyme disease, HIV, leptospirosis, and even multiple sclerosis and/or acute disseminated encephalomyelitis (although the characteristic star exudates are not observed in demyelinating diseases [5, 9, 16]). 

Other ophthalmologic findings related to *Bartonella henselae* infection have been described, such as massive subretinal exudates, multifocal retinochoroiditis, diffuse retinal hemorrhages, vascular occlusive episodes, and necrotizing retinitis. Periodic ophthalmologic examination is mandatory in these cases [9, 15, 17].

Diagnostic suspicion in atypical presentations is difficult, above all in those cases in which there are not any presentations of skin lesions and/or lymphadenopathy, as did occur in this adolescent; this could explain the possibility of delay in diagnosis and treatment [8, 10, 11, 18]. Diagnosis is mainly serological through enzyme immunoassays or indirect immunofluorescence with a high sensibility and specificity [10, 11, 15, 18–20]. This diagnostic possibility makes direct identification and/or microbiology isolation unnecessary. DNA sequencing from gland tissue samples and visceral granulomas require invasive and expensive procedures. In this case, diagnosis was accomplished through serological confirmation by indirect immunofluorescence of an increased IgM antibody titer to *Bartonella henselae* although a significant increase in IgG antibodies titer was observed, enough to consider an acute infection by *Bartonella sp *[10].

Since the disease is fairly benign, azithromycin prescription has been proposed exclusively in those patients with general symptoms or big and/or painful lymphadenopathies as well as immunocompromised patients. Nevertheless, antibiotic therapy is recommended in every patient with an atypical or disseminated presentation of neuroretinitis caused by *Bartonella henselae *despite its evolution is usually benign. The possibility of ophthalmic irreversible structural lesions [5, 17, 18, 21, 22] suggests prescription of combined antibiotic therapy: rifampicin and doxycycline in patients older than eight and rifampicin with azithromycin or co-trimoxazole for patients below that age of four to six weeks [19]. In this patient, antibiotic prescription was based upon clinical and epidemiological suspicion, and steroids were prescribed despite questionable usefulness [9, 15, 16, 22] after the visual evoked potential test findings. Quick and complete recovery of ophthalmic symptoms experienced by this patient, in contrast to other cases referred to by the literature [11, 12, 18], could be in relation to the prompt beginning of treatment.

As a conclusion, cat-scratch disease with associated neuroretinitis is quite rare. It should be suspected in any patient that manifests an abrupt loss of visual acuity together with the finding of disc swelling and macular star exudates.
